# Transcriptomic immaturity of the hippocampus and prefrontal cortex in patients with alcoholism

**DOI:** 10.1038/srep44531

**Published:** 2017-03-15

**Authors:** Tomoyuki Murano, Hisatsugu Koshimizu, Hideo Hagihara, Tsuyoshi Miyakawa

**Affiliations:** 1 Department of Physiological Science, School of Life Science, The Graduate University for Advanced Studies, [SOKENDAI], Kanagawa, Japan; 2 Section of Behavior Patterns, Center for Genetic Analysis of Behaviour, National Institute for Physiological Sciences, Aichi, Japan; 3 Division of Systems Medical Science, Institute for Comprehensive Medical Science, Fujita Health University, Aichi, Japan

## Abstract

Alcoholism, which is defined as the recurring harmful use of alcohol despite its negative consequences, has a lifetime prevalence of 17.8%. Previous studies have shown that chronic alcohol consumption disrupts various brain functions and behaviours. However, the precise mechanisms that underlie alcoholism are currently unclear. Recently, we discovered “pseudo-immature” brain cell states of the dentate gyrus and prefrontal cortex (PFC) in mouse models of psychotic disorders and epileptic seizure. Similar pseudo-immaturity has been observed in patients with psychotic disorders, such as schizophrenia and bipolar disorder. Patients with alcoholism occasionally exhibit similar psychological symptoms, implying shared molecular and cellular mechanisms between these diseases. Here, we performed a meta-analysis to compare microarray data from the hippocampi/PFCs of the patients with alcoholism to data from these regions in developing human brains and mouse developmental data for specific cell types. We identified immature-like gene expression patterns in post-mortem hippocampi/PFCs of alcoholic patients and the dominant contributions of fast-spiking (FS) neurons to their pseudo-immaturity. These results suggested that FS neuron dysfunction and the subsequent imbalance between excitation and inhibition can be associated with pseudo-immaturity in alcoholism. These immaturities in the hippocampi/PFCs and the underlying mechanisms may explain the psychotic symptom generation and pathophysiology of alcoholism.

Alcoholism is one of the major substance abuse disorders and has a lifetime prevalence of 17.8%^1^. Alcoholism is defined by the chronic overconsumption of alcohol and difficulty abstaining from drinking. Both human post-mortem brain studies and animal experiments have indicated that abnormal structural and functional changes play important roles in the pathogenesis of alcoholism^2^,^3^, although the exact mechanisms underlying the development of alcoholism remain unclear. Identifying the molecular and cellular pathological changes in the brains of patients with alcoholism will contribute to the prevention and treatment of alcoholism.

In previous studies, we screened more than 160 mutant mouse strains using a large-scale, comprehensive behavioural test battery^4^ and identified several strains of genetically engineered mice with behavioural traits related to psychiatric disorders^5^,^6^,^7^,^8^. We identified abnormalities by examining the brains of these mice using different methods, especially in the dentate gyrus (DG) of the hippocampus^4^. In these mouse strains, various molecular and electrophysiological aspects of the adult DG neurons were similar to features of the immature DG neurons in normally developing mice, so we named this endophenotype as “immature dentate gyrus (iDG)”^4^,^5^,^6^,^9^,^10^.

We also demonstrated dramatic alterations in the expression of maturity and immaturity markers in the DG neurons of these mice. For instance, the calbindin (a marker of mature granule cells in the DG) gene expression levels were significantly decreased and the calretinin (a marker of immaturity) gene expression levels were significantly increased in these mice. Furthermore, some mice with iDG exhibited increased glial fibrillary acidic protein (GFAP) expression in astrocytes, which is a widely used marker of inflammation. Additionally, decreased parvalbumin and GAD67 expression was observed in the DG in some mice exhibiting iDG^6^. These molecular changes in iDG mice have also been observed in patients with psychotic disorders, such as schizophrenia and bipolar disorder^11^. Previously, we performed genome-wide gene expression pattern analyses and detected transcriptomic immaturity in the prefrontal cortices (PFC) of patients with schizophrenia^6^,^12^. Thus, these immature-like neuronal states appear to exist in various brain areas of patients with psychotic disorders.

Earlier research reported that short-term ethanol exposure reduced the number of calbindin-positive cells but increased the number of GFAP-positive cells in the hippocampal formation^13^. Other studies showed that parvalbumin-positive neurons were damaged and decreased and GFAP-positive cells were increased in an animal model of alcohol abuse^14^,^15^. These molecular marker pattern changes are somewhat similar to the pattern changes observed in mice exhibiting iDG, which suggests that the hippocampus and PFC of ethanol-treated mice may share common transcriptional properties with the immature hippocampus and PFC in normal healthy infants.

The objective of the present study was to address whether immature-like states existed in the alcoholic brain. We performed a meta-analysis using microarray data from different post-mortem studies to comprehensively evaluate the similarities between the hippocampi and PFCs of patients with alcoholism and the hippocampi and PFCs of developing infants. To date, some studies assessing large-scale gene expression in various regions of the alcoholic brain, including the hippocampus and PFC, have been registered in publicly available databases. Thus, we conducted a bioinformatics analysis using these public microarray datasets to investigate whether the maturation states of the hippocampus and PFC were affected in alcoholism. We compared the genome-wide gene expression patterns of the developing human hippocampus/PFC with expression patterns from adult alcoholic hippocampus/PFC samples using microarray datasets from several independent groups. The overlap between gene sets and the directional information for each gene were also examined.

## Materials and Methods

### Data collection and processing

Here, we utilized the following seven publicly available microarray datasets (Table 1): two datasets from the developing human hippocampi and PFCs (GSE25219^16^), two datasets from the human alcoholic hippocampi and PFCs (GSE44456^17^ and GSE49376^18^), and three datasets for mouse cell type-specific gene expression (fast-spiking [FS] neurons [GSE17806^19^], astrocytes [GSE9566^20^], and oligodendrocytes [GSE9566^20^]). All microarray datasets were analysed using the BaseSpace Correlation Engine (BSCE; formally known as NextBio; https://japan.ussc.informatics.illumina.com/c/nextbio.nb; Illumina, Cupertino, CA, USA), which is a microarray experiment database. Researchers can search the database for expression profiles and other results. The data stored in the BSCE undergo several preprocessing, quality control, and organization stages. Quality examinations ensure the integrity of the samples and datasets and include evaluations of pre- and post-normalization boxplots, missing value counts, and *P*-value histograms (after statistical testing) with a false discovery rate analysis to establish whether the number of significantly altered genes is larger than the number anticipated by chance. Additionally, the microarray data were processed with MAS5 (Affymetrix, Santa Clara, CA, USA).

Genes with *P*-values < 0.05 (uncorrected) and absolute fold changes >1.2 were placed in the differentially expressed gene dataset; these parameters represent the lowest sensitivity threshold for commercially available microarray platforms and are the default settings for BSCE analysis. Data from the following Affymetrix GeneChip series were downloaded from the NCBI GEO database: GSE25219, GSE44456, GSE49376, GSE17806, and GSE9566. The Affymetrix Expression Console software (specifically the robust multi-array average algorithm) was used to pre-process the data. We used the expression values (on a log base-2 scale) to calculate the fold changes and *P*-values between two conditions (adults-infants and patients-healthy controls). To determine the fold changes, we divided the infant/disease value by the adult/normal value. Then, these values were converted into the negative reciprocal or -1/(fold change) if the fold change was <1.0. Genes with an absolute fold change >1.2 (PFC: GSE44456, GSE49376, and GSE25219) or >1.3 (hippocampus: GSE25219) and a *t*-test *P*-value < 0.05 were imported into BSCE following the instructions provided by the manufacturer. The signatures in the two gene sets were compared using BSCE. Next, the Running Fisher test^21^, which is a non-parametric rank-based statistical test developed by BSCE, was applied to evaluate the pairwise correlations (overlap) between any two datasets, including datasets from different species and organs^6^,^12^,^22^,^23^. To determine the similarities between the datasets, we also included each gene’s directional information (up- or down-regulation) in the analysis^21^,^22^.

Orthologues for each organism pair were established for comparisons across the various arrays^21^. Information from Mouse Genome Informatics at Jackson Lab (http://www.informatics.jax.org), HomoloGene at NCBI (http://www.ncbi.nlm.nih.gov), and Ensembl (http://www.ensembl.org) was utilized to identify the orthologues. The *P*-value calculated for gene overlap by BSCE indicates a significant association between two sets of genes. The details of these comparisons are provided in Supplementary Information 1. When analysing the overlap between the datasets, the significance level of the *P*-value was corrected for the number of dataset combinations (enumerated in Supplementary Information 1) using the Bonferroni method. Similarly, the significance level for the overlap in directional changes was corrected for the number of possible situations (up, up; down, down; up, down; and down, up) using the Bonferroni method (*P* < 0.0125 = 0.05/4).

The microarray data on FS neurons were obtained from green fluorescent protein (GFP)-expressing neurons isolated by cell sorting from transgenic mice expressing GFP under the control of the GAD1 promoter at postnatal days (P) 7 to 40^19^. To generate the transgenic mice, a 200-kb GAD1 bacterial artificial chromosome fused to a GFP-coding sequence was incorporated into the genome. Collectively, the promoter and integration position limited the GFP expression to a homogeneous subset of gamma-aminobutyric acid (GABA)ergic FS neurons. Thus, in this mouse line, GAD1 promoter-driven GFP expression can be used as a stable marker for potential FS parvalbumin-positive cortical interneurons during development. Here, the results of the analysis comparing the FS neuron data from P40 to the data from P7 were considered to represent the gene expression changes that occurred within this cell type during development.

GFP-positive astrocytes collected by cell sorting from the S100b-GFP transgenic mice at P1–30 were processed for the microarray analysis. The data collected from P1–8 were compared with the data collected from P17–30 to identify gene expression changes that occurred in astrocytes during development^20^.

The oligodendrocytes were purified by antibody-based panning methods using antibodies against maturation markers (platelet-derived growth factor receptor, alpha polypeptide, GalC, and molybdenum cofactor) expressed by the mice at P16 and then processed for the microarray analysis^21^. The microarray data from molybdenum cofactor-positive myelinating oligodendrocytes were compared with the data from GalC-positive pre-myelinating oligodendrocytes; these results were considered to represent the gene expression changes that occurred in oligodendrocytes during development.

However, the different cell collection methods and the different maturation time points used for each cell type should be considered when directly comparing the contributions of the cell types.

## Results

### Gene expression patterns in the hippocampi of patients with alcoholism resemble the patterns in typically developing infants

**Figure 1:**
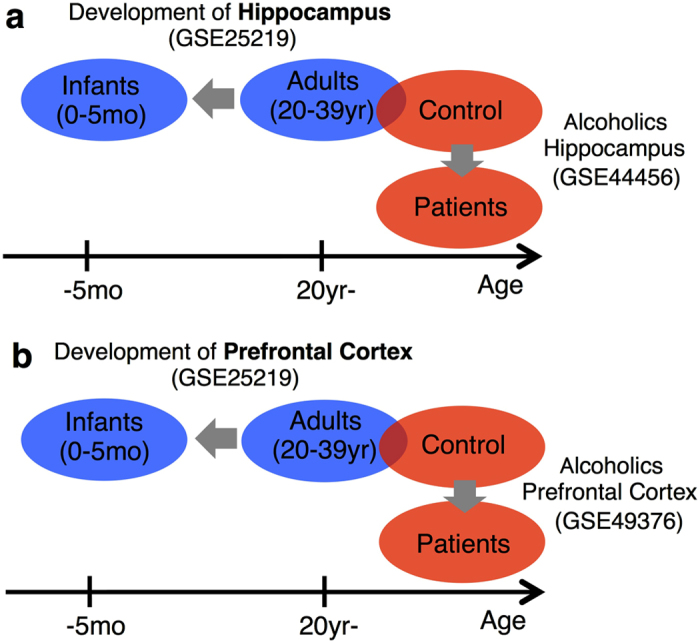
Comparisons of gene expression patterns between the developing and adult alcoholic hippocampus and PFC.

To assess the maturational changes that occurred in the overall gene expression patterns of patients with alcoholism, we compared a microarray dataset from the hippocampi of the patients with alcoholism (GSE44456^17^) to a dataset from the hippocampi of typically developing controls (GSE25219^16^) (Fig. 1a). Non-parametric, rank-based statistical methods that included information about the rank and the direction of the gene expression changes in the overlapping *P*-values were used to analyse the datasets.

**Figure 2:**
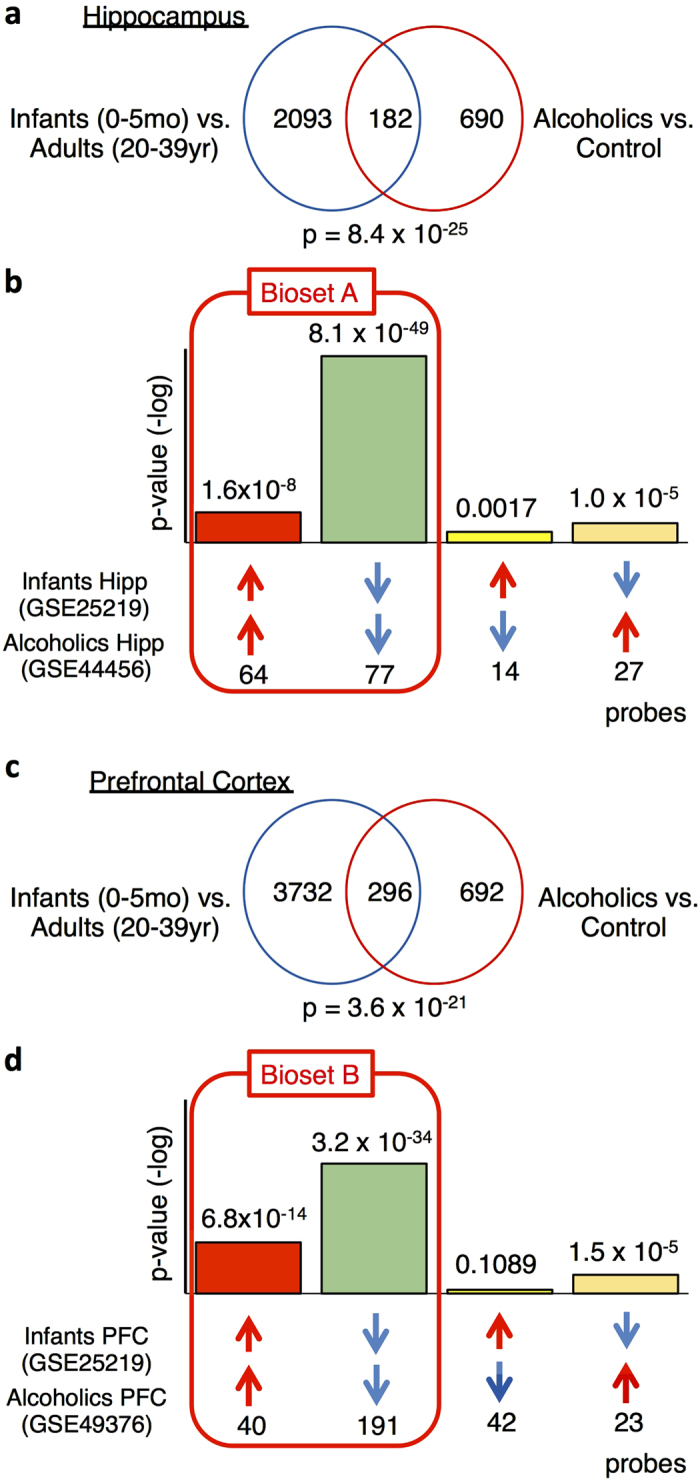
Transcriptomic immaturity of the hippocampus and PFC in patients with alcoholism. (**a**,**c**) Venn diagrams illustrating the overlap in transcriptome-wide gene expression changes in the hippocampi/PFCs of the male patients with alcoholism (patients compared with normal healthy controls [hippocampus: 27–82 years; PFC: 56 ± 10 years]) and typically developing infants (infants: <5 months and adults: 20–39 years). (**b**,**d**) Overlapping *P*-values between the hippocampi/PFCs of the male patients with alcoholism and typically developing individuals (infants: < 5 months and adults: 20–39 years). Bar graphs illustrate the overlapping *P*-value for genes up-regulated (red arrows) or down-regulated (blue arrows) by each condition between the two conditions. Bonferroni correction was used to adjust the significance level according to the number of dataset pairs included in each study (see the Methods section and Additional File 1). Genes that exhibited changes in gene expression in the same direction (i.e., positive correlation) in the two groups in (b) and (d) were designated Biosets A and B (surrounded by red lines), respectively. These Biosets were used in the cell type contribution analysis (Fig. 3).

By querying the database, we found that the gene expression patterns in the hippocampi of the male patients with alcoholism and the typically developing controls strongly overlapped. Of the 872 probes that were altered in the hippocampi of the male patients with alcoholism, 182 probes were also altered during typical hippocampal development, indicating significant similarities in the transcriptomic changes between these two groups (overlapping *P*-value: *P* = 8.4 × 10^−25^; Fig. 2a). Most of the gene expression changes shared between these two groups occurred in the same direction. Among the 182 similarly altered probes, 64 were up-regulated in both groups (*P* = 1.6 × 10^−8^; Fig. 2a), and 77 were down-regulated in both groups (*P* = 8.1 × 10^−49^; Fig. 2a); thus, these probes demonstrated positive correlations. We observed that 14 probes were up-regulated in the infant group but down-regulated in the patient group (*P* = 1.1 × 10^−5^; Fig. 2a), and 27 probes were down-regulated in the infant group but up-regulated in the patient group (*P* = 0.0017; Fig. 2a); hence, these probes exhibited negative correlations.

Collectively, these findings indicate that the overall gene expression patterns in the hippocampi of the male alcoholic patients were highly similar to the patterns in the hippocampi of developing infants. Therefore, these findings suggest that the chronic alcohol consumption induces immature-like gene expression patterns in the hippocampi of the male alcoholic patients. In contrast, the overlapping *P*-value for the comparison between the hippocampi of the female patients with alcoholism and the hippocampi of the typically developing individuals was 0.0003. The microarray data from the female alcoholic patients exhibited less similarity to the data from the developing hippocampi than the data from the male alcoholic patients.

### Gene expression pattern changes in the PFCs of patients with alcoholism are significantly correlated with normal development

The same analysis described above was performed on gene expression in the PFC using different microarray datasets from male patients with alcoholism (GSE49376^18^) and typically developing controls (GSE25219^16^) (Fig. 1b). We observed a significant overlap of the gene expression pattern changes in the PFC between the male patients with alcoholism and the typically developing controls. Of the 988 probes altered in the PFCs of the male patients with alcoholism, 296 probes were also altered in the PFC during normal development; this overlap was significant with a *P*-value of 3.6 × 10^−21^ (Fig. 2b). Most of the shared probes between these two groups were altered in the same direction. Among the 296 shared probes, 40 were up-regulated in both groups (*P* = 6.8 × 10^−14^; Fig. 2b), while 191 were down-regulated in both groups (*P* = 3.2 × 10^−34^; Fig. 2b). In contrast, 42 probes were up-regulated in the infant group but down-regulated in the patient group (*P* = 0.1089; Fig. 2b), whereas 23 probes were down-regulated in the infant group but up-regulated in the patient group (*P* = 1.5 × 10^−5^; Fig. 2b).

Taken together, these results indicated that the gene expression patterns in the PFCs of the male alcoholic patients were highly similar to the patterns in the PFCs of the infants. These findings suggest that chronic alcohol consumption induces immature-like gene expression patterns in the PFCs of the male alcoholic patients. In contrast, the overlapping *P*-value for the comparison between the PFCs of the female patients with alcoholism and the PFCs of the normal infants was 0.0431. The data from the female alcoholic patients exhibited less similarity to the data from the developing PFCs than the data from the male alcoholic patients.

### The contributions of various cell types to the transcriptional immaturity of the hippocampus and PFC in patients with alcoholism

Subsequently, we examined the contributions of several different cell types to the observed immature-like gene expression patterns in the patients with alcoholism. To estimate the contribution of each cell type, we utilized mouse datasets that evaluated developmental gene expression changes in FS neurons, astrocytes, and oligodendrocytes^19^,^20^. Additionally, we selected datasets whose expressions changed in the same direction between human patients with alcoholism and normal human infants (Bioset A from the hippocampus: 141 genes and Bioset B from the PFC: 231 genes). We compared these two gene group types from mice and humans, which represented the development of specific cell types and immaturity in alcoholism, respectively, and determined the overlapping *P*-value between these two groups. The same analyses were performed for both the hippocampus and the PFC.

**Figure 3:**
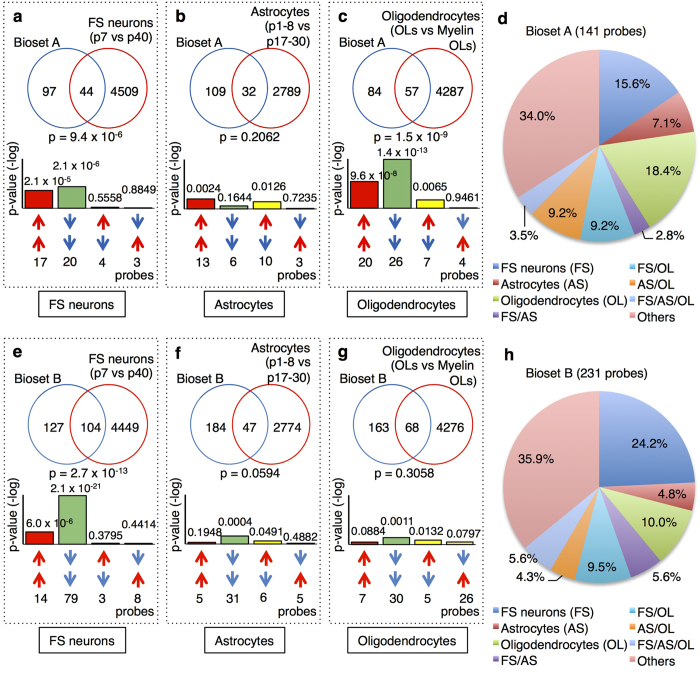
Cell type contributions to Transcriptomic immaturity in the alcoholic hippocampus and PFC. Genes exhibiting changes in expression in the same direction between the normal developing and adult alcoholic hippocampi and PFCs (**a–d**), Bioset A in hippocampus and (**e–h**), Bioset B in PFC) were compared to the gene expression changes obtained from cell type-specific developmental experiments (**a**,**e**), FS neurons [GSE17806], (**b**,**f**) astrocytes [GSE9566], and (**c**,**g**) oligodendrocytes [GSE9566]). Venn diagrams illustrate the overlap in transcriptome-wide alterations in gene expression between the two conditions. Bonferroni correction was used to adjust the significance level according to the number of dataset pairs in each study (see the Methods section and Additional File 1). Bar graphs illustrate the overlapping *P*-values for genes up-regulated (red arrows) or down-regulated (blue arrows) by each condition. Note that the scale of the y-axis is the same in (**a**–**c** and **e**–**g**). **(d**,**h**) Pie charts representing the percentage that each cell type contributed to the altered gene expression in Bioset A and Bioset B. AS, astrocytes; FS, FS neurons; OL, oligodendrocytes.

Of the 141 genes in Bioset A, which contained genes exhibiting positively correlated expression changes between the developing and alcoholic hippocampi, 44 (31.2%) genes were also altered in the datasets for mouse FS neuron development (overlapping *P*-value: *P* = 9.4 × 10^−6^; Fig. 3a). Moreover, 32 genes (22.7%) and 57 genes (40.4%) in Bioset A overlapped with genes in the datasets for mouse astrocyte development (*P = *0.2062; Fig. 3b) and mouse oligodendrocyte development (*P* = 1.5 × 10^−9^; Fig. 3c), respectively. Interestingly, many of the commonly altered genes between Bioset A and the datasets for each cell type exhibited the same directional changes in expression; specifically, a positive correlation was observed for 37 of the 44 genes for mouse FS neuron development and for 46 of the 57 genes for mouse oligodendrocyte development.

Additionally, of the 141 Bioset A genes, 22 (15.6%), 10 (7.1%), and 26 (18.4%) genes exhibited significant developmental changes that were specific to mouse FS neurons, astrocytes, and oligodendrocytes, respectively (Fig. 3d). Four (2.8%), 13 (9.2%), and 13 (9.2%) genes were shared between FS neurons and astrocytes, FS neurons and oligodendrocytes, and astrocytes and oligodendrocytes, respectively (Fig. 3d). Five of the 141 genes (3.5%) in Bioset A exhibited common developmental regulation among the three cell types (Fig. 3d).

Similar trends were found for the contributions of the FS neuron to the immature-like patterns of gene expression in Bioset B, which contained genes with positively correlated expression changes between the developing and alcoholic PFCs. When comparing Bioset B to the developmental data from each cell type, the expression of 104, 47, and 68 genes overlapped with genes expressed during mouse FS neuron (*P* = 2.7 × 10^−13^; Fig. 3e), mouse astrocyte (*P* = 0.0594; Fig. 3f), and mouse oligodendrocyte development (*P* = 0.3058; Fig. 3g), respectively. Among the 104 overlapping genes between Bioset B and the mouse FS neurons, 93 exhibited a positive correlation. Similar to the results obtained for the hippocampal data, most of the PFC genes were altered in the same direction between Bioset B and the mouse FS neuron datasets.

Among the 231 genes in Bioset B, 56 (24.2%), 11 (4.8%), and 23 (10.0%) genes exhibited significant developmental changes that were specific to mouse FS neurons, astrocytes, and oligodendrocytes, respectively (Fig. 3h). In addition, 13 (5.6%), 22 (9.5%), and 10 (4.3%) genes were shared between the FS neurons and astrocytes, FS neurons and oligodendrocytes, and astrocytes and oligodendrocytes, respectively (Fig. 3h). Thirteen of the 231 genes (5.6%) in Bioset B exhibited common developmental regulation in these three cell types (Fig. 3h). In total, the contribution of FS neurons to the immaturity identified in the alcoholic hippocampus and PFC was dominant compared to the contributions of the other cell types examined.

## Discussion

Here, we demonstrated that the genome-wide gene expression profiles of alcoholic hippocampi and PFCs were similar to the hippocampi and PFCs of developing brains. Indeed, the relative gene expression patterns in the hippocampus and PFC of patients with alcoholism (particularly in the male subjects) were significantly similar to the expression patterns of infants according to our bioinformatics analysis. Deficits in various cognitive functions, including long-term memory^24^, working memory^14^,^25^, prospective memory^26^ and executive functions^27^, are often observed in patients with alcoholism and are thought to be associated with hippocampal and PFC dysfunction^28^,^29^. Generally, infants and children perform more poorly than adolescents or adults in working memory^30^ and long-term memory^31^ tasks. We previously reported that immature neurons support the forgetting of remote memories and disrupt the formation of new memories^32^. In addition to these prior findings, our current data imply that the gene expression patterns in the hippocampi and PFCs of the patients with alcoholism resemble the patterns in normal infants, suggesting that the dysfunctions in alcoholic patients could be partially attributed to immature-like abnormalities in the hippocampus and PFC.

We also performed analyses on the contributions of various cell types to the immaturity in the hippocampus and PFC using cell type-specific gene expression data from mice and Running Fisher algorithm which enable us to compare the data from different species. The results showed that FS neuron contribution was most dominant to the immaturity in hippocampus and PFC of patients with alcoholism. The results imply that chronic alcohol exposure changes the maturation status of FS neurons in both the hippocampus and PFC and that the dematuration of FS neurons followed by an imbalance between excitatory and inhibitory circuits may further contribute to the immaturity observed in the hippocampi and PFCs of patients with alcoholism. This idea is consistent with the fact that pilocarpine treatment, which may also cause such imbalance between excitatory and inhibitory circuit, induces dematuration^9^. Because we only analysed the contribution of three cell types to the induction of hippocampal and PFC immaturity, we could not exclude the possibility that other cell types might be involved. An alternative hypothesis that may explain the relationship between alcoholism and impaired FS neuron function is the use of an inverse order. For example, a person who innately has maturational abnormalities in these brain regions will develop down-regulated inhibitory functions in the PFC and reduced top-down signals from the PFC, which may lead to impaired self-regulation^33^,^34^,^35^. These maturational abnormalities would also impair the abilities of the hippocampus and PFC to control dopamine signalling in reward systems^35^,^36^. Indeed, individuals with impaired inhibitory neural functions often fail to self-regulate and stop binge-drinking behaviours, ultimately resulting in alcoholism. These two mechanisms may mutually interact and contribute to the development of alcoholism.

To date, we have detected brain immaturity in various situations and hypothesized that the hyper-excitability of neurons is one of the causes of pseudo-immaturity in the hippocampus and PFC^4^; other reports also support an association between the hyper-excitation of neurons and neuronal dematuration^9^,^10^. In the present study, we detected immaturities in the brains of patients with alcoholism. The results of our analysis suggest that chronic alcohol consumption and the subsequent hyper-excitation of neurons may induce brain immaturity. Ethanol acutely potentiates GABAa receptors, whereas chronic and intermittent alcohol consumption are thought to induce the down-regulation of GABAergic functions^3^,^37^. Indeed, some previous studies have reported the down-regulation of inhibitory neural transmission in alcoholism^14^,^15^. This finding is consistent with the results from our bioinformatics analysis, which suggests that FS neurons are immature in the brains of patients with alcoholism (Fig. 3). The down-regulation of GABAergic functions in alcoholic patients may lead to an imbalance between excitation and inhibition that favours hyper-excitability^3^. Additionally, reports have described seizures and epilepsy in patients with alcoholism^38^,^39^,^40^, indicating that neuron hyper-excitability is present in alcoholism.

Together, these findings support our hypothesis that neuron hyper-excitability may be significantly involved in the aetiology of neuronal dematuration in alcoholism. Thus, alcoholism appears to be associated with the “immature dentate gyrus” and “immature prefrontal cortex” phenotypes, which may be induced by neuronal hyperactivity. Therefore, by focusing on the pseudo-immaturity of the alcoholic brain, the current results may further our understanding of the pathogenesis of alcoholism and aid in the identification of new drug targets for alcoholism.

## Additional Information

**How to cite this article:** Murano, T. *et al*. Transcriptomic immaturity of the hippocampus and prefrontal cortex in patients with alcoholism. *Sci. Rep.* **7**, 44531; doi: 10.1038/srep44531 (2017).

**Publisher's note:** Springer Nature remains neutral with regard to jurisdictional claims in published maps and institutional affiliations.

## Supplementary Material

Supplementary Information

## Figures and Tables

**Table 1:** Microarray datasets used in this study.

GEO accession	Reference	Microarray platform	Sample	No. of samples
GSE25219	Kang *et al*., Nature^16^	GPL5175 Affymetrix Human Exon 1.0 ST Array [transcript (gene) version] GPL5188 Affymetrix Human Exon 1.0 ST Array [probe set (exon) version]	Hippocampus of infants (0–5 mo) and adults (20–39 yr); Orbital PFC of infants (0–5 mo) and adults (20–39 yr)	Hippocampus: infants = 5, adults = 12; PFC: infants = 5, adults = 16
GSE44456	McClintick *et al*., Alcohol^17^	GPL6244 Affymetrix Human Gene 1.0 ST Array [transcript (gene) version]	Hippocampus of male patients with alcoholism	patients = 14, controls = 14
GSE49376	Xu *et al*., Hum Mol Genet^18^	GPL10904 Illumina HumanHT-12 V4.0 expression beadchip (gene symbol)	Prefrontal cortex of male patients with alcoholism	patients = 16, controls = 16
GSE17806	Okaty *et al*., J Neurosci^19^	Mouse430.2.0	Fast-spiking interneurons isolated from the somatosensory cortex of P7 and P40 mice	P7 = 3, P40 = 3
GSE9566	Cahoy *et al*., J Neurosci^20^	Mouse430.2.0	Astrocytes isolated from the forebrains of P1–30 mice and oligodendrocytes isolated based on maturation marker expression from the forebrains of P16 mice	Ast: P1–8 = 4, P17–30 = 4; OL: OL = 4, Myelin OL = 4
