# Supplementary material for: Transcriptomic immaturity of the hippocampus and prefrontal cortex in patients with alcoholism
Source: Sci Rep. 2017 Mar 15;7:44531. doi: 10.1038/srep44531 (PMC5353747; doi:10.1038/srep44531)
Supplement: Supplementary Information [file srep44531-s1.pdf]

Transcriptomic immaturity of the hippocampus and prefrontal cortex in patients with alcoholism

Tomoyuki Murano[1,2,3], Hisatsugu Koshimizu[3], Hideo Hagihara[3], Tsuyoshi Miyakawa[2,3]

[1]Department of Physiological Science, School of Life Science, The Graduate University for Advanced Studies, Japan [SOKENDAI]
[2]Section of Behavior Patterns, Center for Genetic Analysis of Behavior, National Institute for Physiological Sciences, Japan
[3]Division of Systems Medical Science, Institute for Comprehensive Medical Science, Fujita Health University, Toyoake, Japan

*Correspondence: miyakawa@fujita-hu.ac.jp

Supplementary Information

Computing overlap p-value of gene expression patterns from different datasets

NextBio compares the signatures in publicly available microarray data sets with a signature provided by the user using a "Running Fisher" algorithm, as previously described[1]. The overlap *P* value, i.e., the direction of the correlation between two given gene signature sets (*b1*, *b2*), and the *P* values between subsets of gene signatures, is calculated as follows:

First, each gene signature set was rank-ordered according to the absolute fold-change value. Upregulated and downregulated genes were denoted by positive and negative signs, respectively, to imply directionality. A directional subset was generated for each direction, such as *b1+*, *b1-*, *b2+*, and *b2-*. Second, all of the subset pairs were identified as *b1Di*, *b2Dj*, where *Di* and *Dj* were the available directions (+ or -) in *b1* and *b2*, respectively. The Running Fisher algorithm was applied to each subset pair. The top ranking genes in the first subset *b1Di* were collected as a group, *G*, and the second subset *b2Dj* was scanned from top to bottom in rank order to identify each rank with a gene matching a member in group *G*. At each matching rank, *K*, the scanned portion of the second subset *b2Dj* consisted of *N* genes, and the overlap between group *G* and *N* genes was defined as *M*. A Fisher's exact test was performed at rank *K* to evaluate the statistical significance of observing *M* overlaps between a set of size *G* and a set of size *N*, where the set of size *G* comes from platform *P1* and the set of size *N* comes from platform *P2*, given the sizes of *P1* and *P2* as well as the overlap between *P1* and *P2*. At the end of the scan, the best *P* value was retained, and a multiple hypothesis testing correction factor was applied. The negative log of the multiple testing corrected best *P* value ($P_{b1Di \rightarrow b2Dj}$) was a score ($S_{b1Di \rightarrow b2Dj}$) for the subset pair. Here, the subscript of $b1Di \rightarrow b2Dj$ indicates that *b*1*Di* was the first subset used to define the top genes *G* and *b*2*Dj* was the second subset that is used for the scan.

$$S_{b1Di \rightarrow b2Dj} = -\ln P_{b1Di \rightarrow b2Dj} \quad (1)$$

Next, the Running Fisher algorithm was performed in the reverse direction. The same procedure in this reverse direction produced another score ($S_{b2Dj \rightarrow b1Di}$) for the same subset pair. The two scores were averaged to represent the magnitude of the similarity between the two subsets.

$$S_{b1Dib2Dj} = \frac{S_{b1Di \rightarrow b2Dj} + S_{b2Dj \rightarrow b1Di}}{2} \quad (2)$$

The P value ($P_{b1Dib2Dj}$) between $b1Di$ and $b2Dj$ was calculated using the following equation:

$$P_{b1Dib2Dj} = exp\ (-S_{b1Dib2Dj}) \quad (3)$$

A positive sign was assigned to pairwise correlation scores (S_{b1+b2+} and S_{b1-b2-}) for a subset pair of the same direction ($b1+b2+$, $b1-b2-$), and a negative sign was assigned to pairwise correlation scores (S_{b1+b2-} and S_{b1-b2+}) for a subset pair of opposite directions ($b1+b2-$, $b1-b2+$). Then, the overall score (S_{b1b2}) between $b1$ and $b2$ was calculated from the correlation scores (S_{b1+b2+}, S_{b1-b2-}, S_{b1+b2-}, and S_{b1-b2+}) of subset pairs using the following equation:

$$S_{b1b2} = \frac{S_{b1+b1+} + S_{b1-b2-}}{2} - \frac{S_{b1+b1-} + S_{b1-b2+}}{2} \quad (4)$$

The sign of S_{b1b2} determined whether the two signatures were positively or negatively correlated. The overall P value (P_{b1b2}) between $b1$ and $b2$ was calculated using the following equation:

$$P_{b1b2} = exp\ (-|S_{b1b2}|) \quad (5)$$

This overall P value was referred as an overlap P value between two gene expression patterns in this paper.

1. Kupershmidt, I. *et al.* Ontology-Based Meta-Analysis of Global Collections of

High-Throughput Public Data. *PLoS ONE* **5,** e13066 (2010).

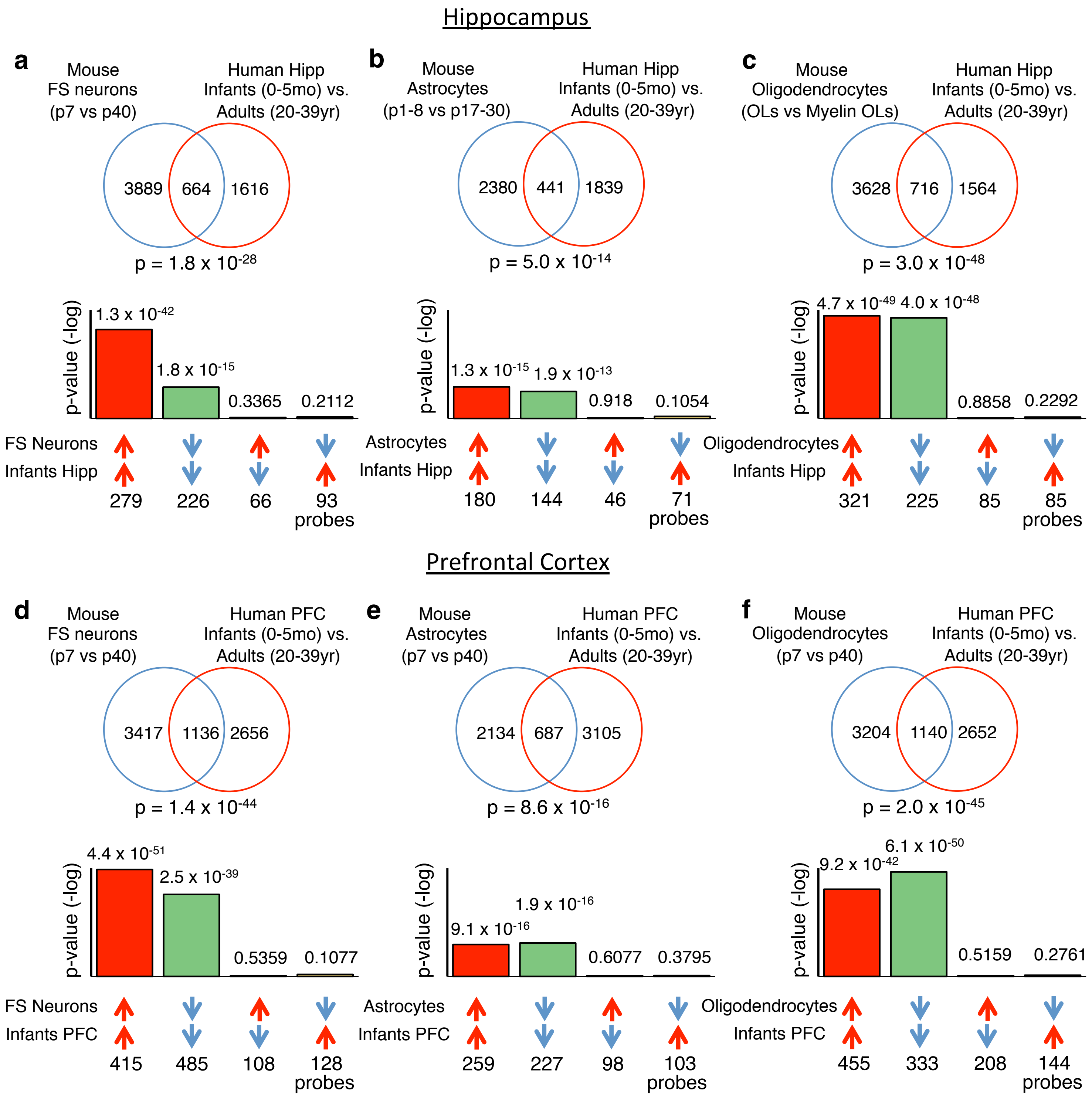
Hippocampus
a
Mouse FS neurons (p7 vs p40)
Human Hipp Infants (0-5mo) vs. Adults (20-39yr)
3889
664
1616
p = 1.8 x 10-28
p-value (-log)
1.3 x 10-42
1.8 x 10-15
0.3365
0.2112
FS Neurons
Infants Hipp
279
226
66
93 probes
b
Mouse Astrocytes (p1-8 vs p17-30)
Human Hipp Infants (0-5mo) vs. Adults (20-39yr)
2380
441
1839
p = 5.0 x 10-14
p-value (-log)
1.3 x 10-15
1.9 x 10-13
0.918
0.1054
Astrocytes
Infants Hipp
180
144
46
71 probes
c
Mouse Oligodendrocytes (OLs vs Myelin OLs)
Human Hipp Infants (0-5mo) vs. Adults (20-39yr)
3628
716
1564
p = 3.0 x 10-48
p-value (-log)
4.7 x 10-49
4.0 x 10-48
0.8858
0.2292
Oligodendrocytes
Infants Hipp
321
225
85
85 probes
Prefrontal Cortex
d
Mouse FS neurons (p7 vs p40)
Human PFC Infants (0-5mo) vs. Adults (20-39yr)
3417
1136
2656
p = 1.4 x 10-44
p-value (-log)
4.4 x 10-51
2.5 x 10-39
0.5359
0.1077
FS Neurons
Infants PFC
415
485
108
128 probes
e
Mouse Astrocytes (p7 vs p40)
Human PFC Infants (0-5mo) vs. Adults (20-39yr)
2134
687
3105
p = 8.6 x 10-16
p-value (-log)
9.1 x 10-16
1.9 x 10-16
0.6077
0.3795
Astrocytes
Infants PFC
259
227
98
103 probes
f
Mouse Oligodendrocytes (p7 vs p40)
Human PFC Infants (0-5mo) vs. Adults (20-39yr)
3204
1140
2652
p = 2.0 x 10-45
p-value (-log)
9.2 x 10-42
6.1 x 10-50
0.5159
0.2761
Oligodendrocytes
Infants PFC
455
333
208
144 probes

Supplementary Figure 1. Strong Correlation between datasets for mouse cell type-specific development and datasets for human hippocampi/PFCs development. Comparison between datasets from each cell type development (**a**, **d**, FS neurons [GSE17806]; **b, e**, astrocytes [GSE9566]; **c, f,** oligodendrocytes [GSE9566]) with datasets from human hippocampi development (**a**, **b, c,** [GSE44456]) and PFCs development (**d**, **e, f,** [GSE49376]). Venn diagrams illustrating the overlap in transcriptome-wide alterations in gene expression in the hippocampi/PFCs of male patients with cell type-specific gene expression. Bar graphs illustrate the overlapping *P*-value of genes up-regulated (red arrow) or down-regulated (blue arrows) by each condition, between the two conditions.
